# An Update on Recent Clinical Trial Data in Bloodstream Infection

**DOI:** 10.3390/antibiotics13111035

**Published:** 2024-11-01

**Authors:** Adam G. Stewart, Peter Simos, Pirathaban Sivabalan, Laura Escolà-Vergé, Katherine Garnham, Burcu Isler

**Affiliations:** 1 Centre for Clinical Research, Faculty of Medicine, The University of Queensland, Royal Brisbane and Women’s Hospital Campus, Brisbane 4006, Australia; 2 Department of Infectious Diseases, Gold Coast University Hospital, Gold Coast 4215, Australia; peter.simos@health.qld.gov.au (P.S.); katherine.garnham@health.qld.gov.au (K.G.); 3 Department of Infectious Diseases, Gosford Hospital, Gosford 2250, Australia; pirathaban.sivabalan@gmail.com; 4 Servicio de Enfermedades Infecciosas, Hospital Universitari Vall d’Hebron, Departamento de Medicina, Universitat Autònoma de Barcelona, 08193 Barcelona, Spain; 5 Pathology Queensland, Gold Coast Hospital Campus, Gold Coast 4215, Australia; 6 Infection Management Services, Princess Alexandra Hospital, Brisbane 4102, Australia; burcu.isler@health.qld.gov.au

**Keywords:** bacteraemia, bloodstream infection, clinical trial

## Abstract

Bloodstream infections (BSIs) remain a significant source of morbidity and mortality globally, exacerbated by an ageing population and rising antimicrobial resistance (AMR). This review offers an updated evaluation of randomized clinical trials (RCTs) in BSI management from 2018 onwards, focusing on the evolving landscape of diagnostics and treatment. New rapid diagnostic technologies and shorter antimicrobial courses have transformed clinical practice, reducing the time to appropriate therapy and hospital stays. Several RCTs demonstrated that rapid phenotypic and genotypic tests shorten the time to optimal therapy, especially when paired with antimicrobial stewardship. Ongoing trials are investigating novel antimicrobial regimens and the safety of early oral switch strategies, particularly for Gram-positive and Gram-negative BSIs. Recent RCTs on *Staphylococcus aureus* BSI (SAB) and multidrug-resistant Gram-negative bacteria highlight advances in treatment but emphasize the need for further study into the efficacy of combination therapies and the utility of rapid diagnostics in different healthcare settings. The review also explores challenges in trail design, with adaptive and pragmatic appropriates improving the efficacy of clinical trials. Finally, this paper identifies gaps in the research, including the need for further investigation into oral step-down therapy, optimal durations, and the role of rapid diagnostics in resource-limited settings.

## 1. Introduction

Bloodstream infection (BSI) is a major cause of morbidity and mortality in both hospital and community settings [1,2]. Both an ageing population and the increasing prevalence of comorbidities, i.e., malignancy, end-organ disease, and diabetes mellitus, contribute to the growing burden of BSIs worldwide [3,4]. Moreover, the global rise in antimicrobial resistance (AMR) has led to more patients receiving inappropriate empirical therapy, and to an increase in overall mortality [5]. More antibiotics have entered the development pipeline than ever before to meet the growing demand imposed by AMR [6]. Advances in microbiological techniques have promised significant improvements in the laboratory processing of blood culture specimens, reducing turnaround time and time to appropriate antibiotic therapy [7]. As more data become available, clinicians are becoming more and more comfortable for shorter durations of therapy and for early oral switch in uncomplicated BSI [8]. Modern clinical trial designs have been adopted (and are currently recruiting) and are equipped to efficiently answer multiple clinical questions in a single trial [9,10]. The purpose of this review is to provide a comprehensive and critical update of RCT data on BSI from 2018 onwards. In addition, we will highlight currently recruiting and planned clinical trials in this area, as well as unmet needs and gaps for further study.

## 2. Laboratory Investigations for Bloodstream Infection

Culture of whole blood in enriched broth media using automated continuous detection systems is the gold standard for the diagnosis and antimicrobial susceptibility testing (AST) of BSI [11]. Limitations comprise potential turnaround times of days for identification and AST results; fastidious, intracellular, or non-bacterial non-yeast pathogens that are undetectable; and reduced sensitivity from pre-analytic practises, including suboptimal specimen volume and antimicrobial administration prior to collection [12]. Consequently, appropriate antimicrobial therapy can be negatively impacted, contributing to patient morbidity and mortality, delayed hospital discharge, and increased healthcare costs. A variety of rapid diagnostic platforms have been developed to reduce the time to results in this critical window, some of which have been the subject of clinical RCTs [7].

Three RCTs have examined the impact of rapid phenotypic AST of blood culture isolates. An in-house method was evaluated by Christensen et al., using the Vitek2 (bioMérieux, France) inoculated with serum separator tube pellet from a positive blood culture bottle [13]. Kim et al. and Banerjee et al. evaluated the commercial QMAC-dRAST (QuantaMatrix, Seoul, Republic of Korea) and Accelerate PhenoTest BC Kit^TM^ (Accelerate Diagnostics, USA), respectively [14,15]. The Accelerate Pheno panel limitations were that 10% of blood culture isolates were not on the panel, and of the 90% which were, there was a 12% identification discordance rate. The panel had 24 (1%) minor AST errors (calling a susceptible antimicrobial resistant) and 2 (0.1%) very major errors (calling a resistant antimicrobial susceptible).

All RCTs observed reduced time to susceptibility results and significantly reduced time to appropriate antimicrobials. Banerjee et al. noted that appropriate antimicrobial changes were more rapidly implemented in the intervention group [15]. Christensen et al. noted that the reduced time to appropriate antimicrobials was during antimicrobial stewardship (AMS) working hours, but not outside of working hours, suggesting that the expert interpretation and implementation of results is key [13]. Kim et al. and Banerjee et al. noted no significant differences in clinical, microbiologic, or cost outcomes [14,15], whilst Christensen et al. observed faster time to oral antimicrobial therapy and shorter hospital stays in the intervention group, with subsequent savings of up to USD 1.2 million [13].

Two RCTs have examined the impact of rapid genotypic identification of pathogen species and resistance determinants. Beuving et al. studied the use of bacterial growth curves combined with antibiotic incubation and quantitative16S PCR for identification and AST, whilst Banerjee et al. evaluated the FilmArray Blood Culture ID panel (BioFire Diagnostics), with or without real-time AMS, against standard processing [16,17]. Both found reduced time to AST results, and also reduced time to appropriate antimicrobial use, most marked in the real-time AMS group. Beauving et al. observed that the adequacy of antimicrobials correlated with reduced length of stay, and Banerjee et al. described a non-statistically significant antimicrobial cost reduction in the intervention group. Neither study observed changes in other clinical outcomes [16,17].

There are two RCTs currently registered with clinicaltrials.gov. RABiT (Vasoo et al.) with plans to evaluate the impact of the BCID panel (Biofire Diagnostics Inc., bioMerieux) coupled with the Rosco Diagnostica ESBL and carbapenemase screening kit on optimal antibiotic use and other relevant clinical and economic outcomes (Clinicaltrials.gov, NCT02743585). The Antibacterial Resistance Leadership Group (ARLG) is currently recruiting for the FAST trial in four countries at the time of writing, a multicenter, multinational RCT evaluating rapid phenotypic AST using REVEAL^TM^ (bioMerieux—metabolomic-based AST) compared with standard laboratory practice in areas with a high prevalence of AMR (Clinicaltrials.gov, NCT06174649) (Table 1). ARLG is also developing the Mastermind-BSI protocol, an adaptable platform to facilitate regulatory approval for new diagnostic tests using a single-patient sample to simultaneously evaluate the performance of multiple different assays [18].

The key clinical trial findings were that rapid diagnostic methodologies reduce the time to results, with subsequent optimal antibiotic therapy, which sometimes translates to reduced overall cost, inpatient bed days, and less acquisition of carbapenemase-resistant Enterobacterales [13,14,15,16]. The largest improvements were observed with real-time AMS involvement for result interpretation and implementation. More commercial platforms are being evaluated world-wide, including in lower-resource settings and regions with a high prevalence of AMR to provide the tools needed to improve diagnosis and care for patients.

## 3. Treatment of Gram-Positive Bacteria

Gram-positive BSIs are an increasingly important cause of BSIs [19]. *Staphylococcus* spp., *Enterococcus* spp., and *Streptococcus* spp. are the most common species of Gram-positive bacteria that cause BSIs. This section will mainly focus on newer approaches and trials regarding the management of *S. aureus* BSI (SAB), and briefly on Enterococcal BSI.

SABs are associated with a 30-day mortality of 20–30% and a 90-day mortality of 15–30% [10,20]. The rates of mortality are higher for methicillin-resistant *S. aureus* (MRSA) compared to methicillin-susceptible *S. aureus* (MSSA) [20,21]. Most patients receive a minimum of two weeks intravenous therapy and remain hospitalized for an average of 20 days [22]. Despite these statistics, <3000 participants have been enrolled in completed RCTs (n = 15) for SAB from 2000 to 2021 [23].

Cheng et al. evaluated the role of synergistic daptomycin (6 mg/kg) for five days in addition to an anti-staphylococcal beta-lactam for MSSA BSIs [24]. This study revealed that synergistic daptomycin did not improve the BSI duration or 90-day all-cause mortality when compared to standard of care. Pacios-Martínez et al. assessed daptomycin and fosfomycin versus daptomycin alone for the treatment of MRSA BSI and infective endocarditis [25]. Daptomycin plus fosfomycin provided 12% higher treatment success than daptomycin alone; however, this was not statistically significant. Furthermore, the combination arm was associated with lower microbiological failure but a higher adverse event profile. Recently, a double-blinded RCT looked at the use of ceftobiprole versus daptomycin with or without aztreonam for complicated SAB (both MSSA and MRSA) [26]. The trial showed that ceftobiprole was non-inferior to daptomycin for the treatment of complicated SAB. However, this trial did not analyze ceftobiprole against methicillin agents for MSSA BSIs or vancomycin for MRSA BSIs; thus, it is difficult to compare this against the standard of practice.

Regarding adjunctive therapies for SABs, no strong evidence has supported their use [24,27]. The ARREST trial analyzed the role of adjunctive rifampicin in the management of SAB; however, no significant overall benefit was noticed compared to standard therapy [27]. Geriak et al. undertook a pilot study RCT investigating the role of combination daptomycin and ceftaroline versus standard monotherapy [28]. The mortality was higher in the monotherapy arm (26%) compared to the combination arm (0%), so study was ceased early given the disproportionate mortality in the monotherapy arm. Given that the study was halted early, the number of study participants in the RCT was low. Grillo et al. evaluated cloxacillin plus fosfomycin versus cloxacillin alone for MSSA BSI, and there was no benefit to the combination arm when assessing treatment success at 7 days [29]. Several guidelines suggest the use of anti-toxin antibiotics such as clindamycin or linezolid where there is a high burden of diseases [30,31]. However, given the toxicities associated with these additional agents, more trials need to be undertaken to justify their use. The CAMERA-2 study evaluated the use of adjunctive anti-staphylococcal beta-lactam for seven days with either vancomycin or daptomycin for MRSA BSI [32]. The rate of BSI by Day 5 into therapy was less in the combination arm; however, there was no difference in the 90-day mortality, relapse, or treatment failure between the two groups. The trial was terminated early, secondary to the increased nephrotoxicity seen in the combination arm compared to the monotherapy arm; however, this was observed with anti-staphylococcal penicillin rather than cefazolin [32].

Questions still remain on the best antibiotic for SAB treatment, the role of adjunctive therapy, on whether early oral switch is safe, and on the role of PET-CT in the management of SAB. These questions will hopefully be answered in the current *Staphylococcus aureus* Network Adaptive Platform (SNAP) trial, which is an adaptive platform trial that involves >7000 adult and child participants. Given the trial design, multiple domains can be answered in parallel [10].

Enterococcal BSIs are the fourth leading cause of BSIs. Despite this, there is a scarcity of evidence, especially RCTs, regarding the management of enterococcal BSIs [33]. The INTENSE study is a multicentre, open-label, RCT assessing the non-inferiority of a 7-day vs. 14-day course for the treatment of uncomplicated enterococcal BSIs and incorporating the early switch to oral antibiotics when feasible. It is currently in progress and will hopefully provide more robust evidence regarding the management of *Enterococcus* species BSIs [34].

## 4. Treatment of Gram-Negative Bacteria

The MERINO trial, an international multicentre RCT, compared piperacillin–tazobactam against meropenem for treating BSIs caused by ceftriaxone-resistant *Escherichia coli* or *Klebsiella pneumoniae*, aiming to assess if piperacillin–tazobactam could serve as a carbapenem-sparing option [35]. The trial was halted due to harm and futility, as it failed to demonstrate non-inferiority; the 30-day mortality rate was significantly higher in the piperacillin–tazobactam group (12.3%) compared to the meropenem group (3.7%). Despite the theoretical benefits of beta-lactam/beta-lactamase inhibitor combinations in vitro, trial outcomes have highlighted the limitations of piperacillin–tazobactam in treating severe infections caused by ESBL-producing bacteria [36,37,38]. Criticisms of the trial include the absence of extended or continuous infusions of beta-lactam antibiotics, mortality unrelated to the primary BSI, disparity in illness severity between treatment groups, and uncertainty surrounding the adequacy of source control [39,40,41,42]. Finally, the gradient test method used for susceptibility testing is unreliable for piperacillin–tazobactam [43]. A post hoc analysis performed on blood culture isolates from the MERINO trial found that many isolates were in fact piperacillin–tazobactam-non-susceptible using the reference method of broth microdilution (BMD), and many co-harboured narrow spectrum oxacillinases (such as *bla*_OXA-1_) and ESBL [44]. Subsequent re-analysis of the 30-day mortality in patients with piperacillin–tazobactam-susceptible isolates using the European Committee on Antimicrobial Susceptibility Testing (EUCAST) and Clinical Laboratory Standards Institute (CLSI) breakpoints resulted in attenuation of the effect on mortality [43,45].

MERINO-2 was a pilot international, multicentre, open-label RCT comparing piperacillin–tazobactam and meropenem for the definitive treatment of BSIs caused by Gram-negative bacteria with chromosomally encoded AmpC beta-lactamases (*Enterobacter* spp., *Enterobacter aerogenes*, *Serratia marcescens*, *Providencia* spp., *Morganella morganii*, or *Citrobacter freundii*) [46]. A higher proportion of patients assigned to piperacillin–tazobactam met the primary composite outcome (30-day mortality, ongoing fever and/or leucocytosis, and microbiological failure or relapse) compared to meropenem (29% versus 21%), although this was not statistically significant. The findings of the trial are limited by the small number of patients (only 79 of the 850 patients screened were randomized) included, cross-over between groups regarding the empiric therapy used, lack of documentation on adequate source control, and the use of intermittent as opposed to extended or continuous infusions of beta-lactam therapy.

CREDIBLE-CR was a randomized, open-label trial in which adults with a diagnosis of hospital-acquired pneumonia (HAP), ventilator-associated pneumonia (VAP), healthcare-associated pneumonia, BSI or sepsis, or complicated UTIs (cUTI) with evidence of carbapenem-resistant Gram-negative infection were assigned to cefiderocol or the best available therapy (BAT) [47]. Clinical cure was similar in both treatment groups for HAP, VAP, HCAP, and BSI or sepsis. Mortality at Day 14 was higher in patients treated with cefiderocol than in those treated with BAT for pulmonary infections and BSI, but not for patients with cUTI. The higher all-cause mortality between the groups was mostly driven by *Acinetobacter* spp. infections. In fact, 46% of all infections in CREDIBLE-CR were secondary to carbapenem-resistant *Acinetobacter baumannii* [48]. Patients in the cefiderocol group with *Acinetobacter* spp. infections were also more likely to have moderate or severe renal dysfunction, ICU at randomisation, ongoing shock, or shock within 31 days before randomisation. Fifteen percent of patients in the cefiderocol group with carbapenem-resistant isolates showed a significant increase in minimum inhibitory concentration (MIC) values, suggesting potential undetected heteroresistance [49]. However, a post hoc analysis found no correlation between infections defined as heteroresistant and clinical outcomes [50].

The AIDA and OVERCOME trials were multicentre RCTs comparing colistin monotherapy to colistin–meropenem combination therapy for the treatment of severe infections caused by carbapenem-resistant Gram-negative bacteria [51,52]. The basis for the trials was due to the potent in vitro synergy demonstrated when colistin is combined with carbapenems and how combination therapy reduces the emergence of resistance [53]. Both the AIDA and OVERCOME trials included patients with pneumonia and/or BSI, and the most common pathogen identified was *A. baumannii* (77% versus 78%). Although meropenem was given at different doses (2 g every 8 h as an extended-infusion over 3 h in the AIDA trial; 1 g every 8 h as a 30 min infusion in the OVERCOME trial), both trials failed to demonstrate a difference in 28-day mortality between colistin monotherapy and combination therapy.

In the RESTORE-IMI 1 and 2 trials for imipenem–relebactam, favourable clinical responses were observed, although only 6.5% and 5.8% of patients had BSI, respectively [54,55]. The ASPECT-NP trial that demonstrated non-inferiority of ceftolozane–tazobactam for the treatment of nosocomial pneumonia had only 6.1% of trial participants with BSI [56]. The REPROVE trial, which demonstrated the non-inferiority of ceftazidime–avibactam for nosocomial pneumonia, had 4.7% of those with BSI enrolled [57]. Monotherapy with meropenem–vaborbactam demonstrated increased clinical cure, decreased mortality, and reduced nephrotoxicity in the TANGO II trial, although only 47 patients were enrolled, with 46.8% having BSI [58]. The REVISIT study comparing aztreonam–avibactam to meropenem for the treatment of serious infections caused by Gram-negative bacteria demonstrated support for its use in complicated intra-abdominal infection and nosocomial pneumonia [59]. Unfortunately, only 6% of patients in this trial had a baseline BSI.

There are several new beta-lactam/beta-lactamase inhibitor combinations that have recently completed or are currently being evaluated in clinical trials, including aztreonam–avibactam, sulbactam–durlobactam, ceftazidime–avibactam, ceftolozane–tazobactam, imipenem–relebactam, and cefiderocol (Clinicaltrials.gov; NCT03329092, NCT05258851, NCT04882085, NCT04673175).

## 5. Candidemia Trials

Since the early 2000s, when echinocandins were introduced for treating candida BSIs, and following the conducting of RCTs comparing various echinocandins in the late 2000s, there have been relatively few RCTs in this area [60]. Most recent candidemia RCTs conducted since then have happened within the last 5 years and include the trials of the new antifungals rezafungin, isovuconazole, ibrexifungerp, and fosmanogepix in comparison to echinocandins.

Rezafungin, a novel echinocandin, represents the first significant progress in treating candidemia and invasive candidiasis in over 15 years, with notable pharmacokinetic advantages such as a long half-life and higher plasma concentrations than its predecessors. Its effectiveness and safety were evaluated in the STRIVE and ReSTORE trials [61,62]. The STRIVE trial, a phase II study with 207 patients, tested rezafungin against caspofungin. It found clinical and microbiological cure rates of 60.5–76% for weekly rezafungin (400 mg) and 67% for caspofungin [61]. The phase III ReSTORE trial, which compared rezafungin to caspofungin with a 20% non-inferiority margin, faced recruitment challenges, yet managed to enrol 199 patients. The results indicated that rezafungin achieved non-inferiority to caspofungin, with mortality rates of 24% for rezafungin and 21% for caspofungin. Despite these findings, the broad non-inferiority margin and difficulties in recruiting participants highlight the need for a nuanced interpretation of rezafungin’s comparability to caspofungin [62]. The previously demonstrated faster resolution of blood culture positivity was not confirmed in this study, but a signal towards earlier mycological eradication at Day 5 was observed in the rezafungin arm. A pooled analysis of both trials showed similar day 30 all-cause mortality rates (19%) for both treatment arms [63]. Additionally, rezafungin is being investigated in the ReSPECT trial, a phase III prophylaxis study, to compare its efficacy with standard antimicrobial regimens in preventing invasive fungal disease, with results pending for final outcomes (Clinicaltrials.gov, NCT04368559).

Isavacuonazole is a newer azole with a better safety profile as compared to caspofungin in the phase III ACTIVE trial for treating candidemia or invasive candidiasis, involving 450 adults [64]. The primary endpoint was to assess the overall response at the end of intravenous therapy. Isavuconazole’s response rate was 60.3% lower than caspofungin’s 71.1%, failing to meet the non-inferiority margin of 15%. Specifically, isavuconazole had lower response rates in candidemia (64.7% vs. 72.4% for caspofungin) and invasive candidiasis (34.5% vs. 65.8%). This reinforces earlier findings that echinocandins generally outperform azoles in these infections [60,65].

Ibrexafungerp, a new orally available beta-glucan synthase inhibitor effective against echinocandin-resistant fungal strains, was assessed in a phase II randomized trial for the treatment of invasive candidiasis [66]. In this small study, 22 patients were randomized to receive either 500 mg or 750 mg of ibrexafungerp, or standard of care with fluconazole or micafungin for a median duration of 12 days. The results indicated a favourable response in 77% of patients at the end of therapy, but this decreased over time. The company behind ibrexafungerp (SCYNEXIS) recently announced positive interim data from its ongoing phase III FURI and CARES single-arm studies, underscoring the effectiveness of oral ibrexafungerp in treating severe fungal infections (Clinicaltrials.gov; NCT03059992, NCT03363841). It is, however, important to note that these are non-randomized controlled trials, focusing on patients who were either intolerant to standard antifungal therapy or had infections that were refractory to previous antifungal treatment, including those caused by *Candida auris*. As we await further results, the complexity of effectively studying treatments for candidemia becomes increasingly apparent.

Fosmanogepix, a novel antifungal currently in phase III trials, demonstrates effectiveness against difficult fungi like *C. auris* and *Aspergillus fumigatus* [67]. In a phase II trial, it was tested as a first-line treatment for non-neutropenic patients with candidemia. The results showed treatment success in 16 out of 20 patients and a 30-day survival rate of 18 out of 21 patients, as reported by Amplyx. An open-label study of fosmanogepix for the treatment of patients with candidemia/invasive candidiasis caused by *C. auris* (Clinicaltrials.gov, NCT04148287) is currently enrolling participants (Clinicaltrials.gov, NCT04148287). Amongst the planned trials of fosmanogepix is a safety/efficacy study that will investigate fosmanogepix compared with IV caspofungin followed by oral fluconazole in patients with candidemia and/or invasive candidiasis (Clinicaltrials.gov, NCT05421858).

## 6. Comparing Duration and Route of Administration of Antimicrobials for Bloodstream Infection

Yahav et al. performed a non-inferiority randomized, multicentre, open-label trial in which stable inpatients with Gram-negative BSIs were randomized to receive 7 days (n = 306) or 14 days (n = 298) of covering therapy [68]. In the study, 68% of the sources were urinary, 24.5% of patients received immunosuppressant drugs, Enterobacterales represented 90% of the isolates, and 18% were multidrug-resistant. The primary outcome at 90 days (all-cause mortality, relapse of BSI, suppurative or distant complications, readmission or extended stay) occurred in 45.8% in the 7-course group vs. 48.3% in the 14-course group (risk difference, −2.6% [95% CI, −10.5% to 5.3%]), with a non-inferiority margin of 10%. There were no significant differences in adverse events, but a more rapid return to baseline functional status was found in the short-course therapy group.

Molina et al. published a similar randomized trial comparing a 7- (n = 119) versus 14-day-course (n = 129) of antibiotics for the treatment of Enterobacterales BSIs [69]. The study also included outpatients (20%); 10% were immunosuppressed, 13% had a septic shock at presentation, and 15% of isolates were Enterobacterales resistant to cephalosporins. Similarly to the Yahav et al. trial, the predominant sources were urinary and biliary, which may have influenced the success of the trial. The non-inferiority margin of 10% was met for most clinical outcomes (cure and relapse of BSI at 28 days after treatment cessation), except for the relapse of fever (−0.2%, 95%CI −10.4 to 10.1). The study was not designed to assess the risk of adverse events, but the authors found a trend towards an increased risk in the 14-day arm. For this reason, they performed a DOOR/RADAR analysis to accurately balance the benefits of the study (short antibiotic duration) with other potential harms (side effects, relapsing fever, not cure or death) [70]. The DOOR/RADAR showed that patients who received a shorter course of antibiotics had a 77.7% probability of achieving better results than a longer course (more clinical cure with less antibiotic exposure and adverse events). A health-economic study could probably show even more benefit.

A Swiss multicentre non-inferiority point-of-care trial (PIRATE) randomized clinically stable inpatients with Gram-negative BSIs on Day 5 of covering therapy to an individualized CRP-guided antibiotic treatment duration (n = 170), a fixed 7-day duration (n = 169), or a 14-day treatment duration (n = 165) [71]. The clinical failure rate at Day 30 (recurrent BSI or local or distant complications or restarting directed antibiotic treatment or death) occurred in 2.4% patients in the CRP arm (with a median antibiotic duration of 7 days), in 6.6% in the 7-day arm, and in 5.5% in the 14-day arm. Differences between the CRP-arm and the 7-day arm treatments compared to the 14-day arm met the non-inferiority criterion of 10%, but adherence to the CRP strategy and follow-up was not performed in 23% of patients, making interpretation of the results difficult. In addition, no cost-effectiveness analysis was made. A prospective cohort study that included patients from the PIRATE study analyzed if shorter courses of antibiotics were associated with a reduction in detectable antibiotic-resistance genes in the intestinal microbiome [72]. However, they found that the reduction in antibiotic duration by half did not result in a decreased abundance of antibiotic-resistance genes at 30 days and did not improve microbiota species diversity.

The BALANCE trial that also compared 7 versus 14 days has finalized recruitment, but the results have not been published yet [73]. There is also a planned substudy focusing on bacteraemic urinary sepsis [74], and there is another trial that is currently recruiting that will also evaluate 5 days versus 7 days of antibiotics for Gram- negative BSIs from a urinary source [75].

Even though there is good-quality evidence for the safety of 7 day therapy for Enterobacterales BSIs, this practice has not been fully adopted [76]. Patients included in the clinical trials are very selected (mostly urinary and biliary infections, rapid clinical response, monomicrobial BSI, source controlled, not immunosuppressed). In fact, less than 20% of all screened patients are finally randomized. And even in the case of patients that are similar to the ones included in the clinical trials, a shorter duration is not usually chosen. The barriers to implementing this evidence are probably multiple (not enough knowledge, lack of confidence, fear of recurrence, etc.) and should be deeply analyzed.

Finally, there are other two on-going pragmatic trials that evaluate antibiotic duration in different clinical scenarios, the INTENSE trial [25] and the SHORTEN-2 trial on *Pseudomonas aeruginosa* BSI (7 versus 14 days) [76]. However, the recruitment will be probably difficult (*Enterococcus faecalis* BSI is now a major criteria for endocarditis, and many patients with *P. aeruginosa* BSI will be neutropenic).

There is a recent multicentre, open-label, randomized clinical trial that evaluates the safety and efficacy of switching from intravenous to oral antimicrobial therapy in adult patients with Enterobacterales BSIs after 3–5 days of microbiologically active IV therapy [77]. Sixty percent of cases had a urinary source, and sixteen percent of isolates were ESBL-producers. The most frequent oral agents used (chosen by the treating physician) were cephalosporins (35%), beta-lactam/beta-lactamase inhibitor combinations (30%), fluoroquinolone (19%), and trimethoprim–sulfamethoxazole (16%). Treatment failure at 90 days (death, need for additional antimicrobial therapy, microbiological relapse, or infection-related re-admission) occurred in 21/82 (25.6%) in the IV arm, and 18/83 (21.7%) in the oral arm (risk difference −3.7%, 95% CI −16.6% to 9.2%), with a non-inferiority margin set at 10%. The median length of hospital stay was significantly shorter in the oral group (6 days vs. 9 days), but the median duration of antimicrobial therapy was longer (14 vs. 11 days), which probability reflects the need for an antimicrobial stewardship to avoid this.

There is another ongoing randomized non-inferiority clinical trial, the INVEST trial, evaluating an early oral stepdown therapy to oral fluoroquinolone or trimethoprim-sulfamethoxazole in uncomplicated Gram-negative BSIs, and, as is crucial in this type of study, a health economic evaluation will be conducted [34]. We still need new trials to address the question of the best oral regimen for Gram-negative BSIs (highly bioavailable oral beta-lactams versus quinolones or trimethoprim–sulfamethoxazole for Gram-negative BSIs for example) [77].

## 7. New Clinical Trial Design and Knowledge Gaps Requiring Prioritization for Future Bloodstream Infection Trials

Research questions and the design of clinical trials in infectious diseases have undergone significant change in recent years. Moreover, industry-led registration clinical trials of new antimicrobials have also undergone change, mostly in accordance with regulatory body guidance (e.g., U.S. Food and Drug Administration, European Medicines Agency). There remains no regulatory body guidance document on the design and evaluation of new antimicrobials in clinical trials for BSI- or pathogen-directed studies [9]. Rising rates of severe infection caused by antimicrobial-resistant pathogens globally has been a significant driver of both the way in which we design, and the total number of clinical studies for these conditions. Moreover, the way healthcare is delivered has changed dramatically, with the number of outpatient or at-home care options increasing, with emphasis being placed on reduced hospital length of stay. RCTs remain the gold standard in evaluating the true effectiveness of an intervention by minimizing bias and establishing causality [78].

Clinical trial design has undergone several key changes of note. There has been a shift towards a more pragmatic design with less restrictive eligibility criteria and overall participant management matching that of usual patient care [79]. The incorporation of Bayesian adaptive randomisation, where patient outcome data influence the randomisation of future clinical trial patients, offers a faster and more efficient way to answer the of question of intervention efficacy [80]. Multi-arm clinical trial designs have also improved efficiency with the simultaneous evaluation of multiple interventions [81]. The incorporation of rapid diagnostics has enabled the earlier enrolment of trial participants [9]. A review at how we measure participant success in BSI trials has also shifted. Composite endpoints that include both clinically relevant and patient-centred outcomes are integral to the successful translation of trial results into the clinical space [82]. The comparison of such outcomes through DOOR/RADAR or the WIN ratio have been advocated [83]. Moreover, the inclusion of a cost–benefit or economic analysis has also become crucial in the setting of rising healthcare costs globally [84].

Solutions to the many challenges encountered, with regard to pathogen-directed BSI clinical trials, are yet to be determined. Trials need to improve participant eligibility and embed research infrastructure into routine clinical care. This was exemplified in the ACORN randomized clinical trial, whereby within the electronic health record, patients were screened, enrolled, randomized, and assigned a trial antibiotic, with trial data collected within routine care [85]. Trial participants need to mirror real-world patients, such as those who may harbour infection due to a multidrug-resistant pathogen, or those with concomitant comorbidity, who might be historically excluded from clinical trials. A simplified, more streamlined informed consent process, alongside improving site investigator buy-in, has also been advocated for [86]. Early enrolment into pathogen-directed clinical trials is also problematic, with most clinical laboratories relying on slow culture-based techniques for identification and susceptibility testing. The use of new rapid diagnostics for BSIs offers a potential solution to this problem, although this can be cost-prohibitive [12].

The journal Clinical Microbiology and Infection recently published a series of commentary articles on “Which randomized controlled trial do we need?” [87]. This has been in response to a need for more robust data to support common clinical decisions, which often suffer from a lack of evidence on infectious diseases. Many trials have been proposed, including the use of oral antimicrobials for Gram-negative BSIs, definitive therapy for *Pseudomonas aeruginosa* BSIs, and next-generation sequencing to individualize therapy in *Staphylococcus aureus* BSI. This just highlights a few of the evidence gaps in diagnosis and management. The utility and cost-effectiveness of new rapid diagnostics for BSIs require ongoing evaluation in clinical trials. Determining optimal therapy in pathogen-directed trials targeting the WHO Priority Pathogen List (e.g., ESBL, CPE) is a necessity. Duration and oral switch studies for *Staphylococcus aureus* BSIs are also of high priority.

## 8. Conclusions

BSIs are recognized as an important and common clinical entity requiring a strong evidence base for their management. Buoyed by a growing interest in pathogen-directed clinical trials and the ever-rising burden of AMR, more and more clinical trials on BSIs are being undertaken to fill these evidence gaps. Both industry- and investigator-led trials have adapted to improve clinical trial design to enable meaningful and translational trial results. The funding and development of apt trial infrastructure remain a huge hurdle for success, with many planned clinical trials not seeing completion. Continued improvement in trial design and implementation will hopefully see improved completion success, with clinical trial data informing up-to-date guidelines and improving patient care.

## Figures and Tables

**Table 1:** Examples of planned, actively recruiting and recently completed clinical trials for bloodstream infection.

Trial Reference	Trial	N	Sponsor	Population	Countries Recruiting From	Intervention	Outcome	Year Commenced Recruiting	Planned Completion Date
**NCT06174649**	FAST	900	Duke University	Hospitalized subjects with blood cultures growing Gram-negative bacilli	Greece India Israel Spain	SOC AST versus AST using REVEAL™	Composite 3-category DOOR outcome (unsuccessful discharge, lack of clinical response, and undesirable events) plus 30-day mortality	December 2023	May 2025
**NCT05137119**	SNAP	8000	University of Melbourne	Staphylococcus aureus complex grown from ≥1 blood culture	Australia Canada Israel Netherlands New Zealand Singapore South Africa United Kingdom	Platform trial design with silos (PSSA, MSSA, MRSA) and domains (backbone antibiotic, adjunctive antibiotic, early oral switch)	All-cause mortality at 90 days after platform entry	February 2022	December 2028
**NCT05394298**	INTENSE	284	Fundación Pública Andaluza para la gestión de la Investigación en Sevilla	Hospitalized adult patients with monomicrobial E. faecalis or E. faecium bacteremia.	Spain	Non-inferiority of a 7-day antibiotic regimen vs. 14 days in the treatment of bacteremia	Clinical success defined as (a) survival at TOC; (b) absence of enterococcal bacteremia relapse or infective endocarditis diagnosis at TOC; (c) no need to prolong therapy beyond the pre-established duration, or restart drugs against enterococci for any reason within 30 days.	July 2022	July 2024
**NCT03869437**	GAMECHANGER	513	University of Queensland	Hospital acquired or healthcare associated Gram-negative bloodstream infection	Australia Malaysia Singapore Taiwan Thailand Turkey	Cefiderocol versus best available therapy	All-cause mortality at 14 days	October 2019	November 2023
**NCT05421858**	-	450	Basilea Pharmaceutica	Adults with candidemia and/or invasive candidiasis based on a blood or non-blood specimen obtained within ≤96 h (4 days) before randomizatio	-	Oral fosmanogepix versus IV caspofungin followed by oral fluconazole	All-cause mortality at 30 days	August 2024	January 2028
**NCT05210439**	SHORTEN2	306	Fundación Pública Andaluza para la gestión de la Investigación en Sevilla	Adults with BSI-PA who have received 6 days of active antibiotic treatment	Spain	7 versus 14 days of treatment	Probability of achieving better DOOR/RADAR score for patients in the experimental group than in the control group	April 2022	June 2025
**NCT05199324**	INVEST	720	Tan Tock Seng Hospital	Adults with clinically stable/non-critically ill inpatients with uncomplicated Gram-negative bacteraemia	Singapore Australia Malaysia South Korea Turkey Israel Italy Greece Spain Lebanon	Early step-down to oral antibiotics (within 72 h from index blood culture collection) versus continuing standard of care IV therapy (for at least another 24 h post-randomisation)	All-cause mortality at day 30 post-randomisation	April 2022	March 2025

## Data Availability

The original contributions presented in the study are included in the article; further inquiries can be directed to the corresponding author.
